# A Clue to the Existence of Bonding between Bone and Implant Surface: An In Vivo Study

**DOI:** 10.3390/ma12071187

**Published:** 2019-04-11

**Authors:** Taek-Ka Kwon, Jung-Yoo Choi, Jae-Il Park, In-Sung Luke Yeo

**Affiliations:** 1 Division of Prosthodontics, Department of Dentistry, St. Catholic Hospital, Catholic University of Korea, Suwon 16247, Korea; tega95@naver.com; 2 Dental Research Institute, Seoul National University, Seoul 03080, Korea; jychoi55@snu.ac.kr; 3 Animal Facility of Aging Science, Korea Basic Science Institute, Gwangju 61186, Korea; jaeil74@kbsi.re.kr; 4 Department of Prosthodontics, School of Dentistry and Dental Research Institute, Seoul National University, 101 Daehak-ro, Jongro-gu, Seoul 03080, Korea

**Keywords:** osseointegration, titanium, bone–implant interface, shear strength, torque

## Abstract

We evaluated the shear bond strength of bone–implant contact, or osseointegration, in the rabbit tibia model, and compared the strength between grades 2 and 4 of commercially pure titanium (cp-Ti). A total of 13 grades 2 and 4 cp-Ti implants were used, which had an identical cylinder shape and surface topography. Field emission scanning electron microscopy, X-ray photoelectron spectroscopy, and confocal laser microscopy were used for surface analysis. Four grades 2 and 4 cp-Ti implants were inserted into the rabbit tibiae with complete randomization. After six weeks of healing, the experimental animals were sacrificed and the implants were removed en bloc with the surrounding bone. The bone–implant interfaces were three-dimensionally imaged with micro-computed tomography. Using these images, the bone–implant contact area was measured. Counterclockwise rotation force was applied to the implants for the measurement of removal torque values. Shear bond strength was calculated from the measured bone–implant contact and removal torque data. The *t*-tests were used to compare the outcome measures between the groups, and statistical significance was evaluated at the 0.05 level. Surface analysis showed that grades 2 and 4 cp-Ti implants have similar topographic features. We found no significant difference in the three-dimensional bone–implant contact area between these two implants. However, grade 2 cp-Ti implants had a higher shear bond strength than grade 4 cp-Ti implants (*p* = 0.032). The surfaces of the grade 2 cp-Ti implants were similar to those of the grade 4 implants in terms of physical characteristics and the quantitative amount of attachment to the bone, whereas the grade 2 surfaces were stronger than the grade 4 surfaces in the bone–surface interaction, indicating osseointegration quality.

## 1. Introduction

Modern endosseous dental implants have been used widely in dental practice since the Toronto conference in 1982 [1,2]. Modern implants have demonstrated reliable longevity and success, and have become a routine dental therapeutic protocol in edentulous patients. However, the bonding mechanism between the bone and the dental implant is still unclear.

Bone-to-implant contact (BIC) in dental implantology is defined as the direct attachment of bone to an implant observed on an undecalcified histologic slide under a light microscopic view without intervening soft tissue. The nature of BIC, whether a real bond exists between the bone and implant or only simple contact, remains unknown [3]. BIC has been suggested to be hard tissue encapsulation, or the bony isolation of the osseointegrated dental implant [4,5,6]. If such a foreign body reaction is the case, it is highly possible that BIC should be a simple physical attachment between the bone and implant. Therefore, if the implants have identical physical features, such as surface topography and implant design, the shear bond strength or removal torque per unit area of implant would be similar, regardless of the material compositions on implant surfaces. Conversely, different bond strengths imply osseointegration quality at the interface, which suggests the bone has biologic affinity depending on materials, rather than the bony isolation. To the best of our knowledge, no calculation of the interfacial binding per unit area has been published to test whether BIC is only frictional or has its own quality.

This study was designed to test the hypothesis that no significant difference would be found in the removal torque per unit of bone contact area, calculated by micro computed tomography (CT), in a comparison of interfacial bindings composed of grades 2 and 4 commercially pure titanium (cp-Ti), if BIC is a simple contact between the bone and implant. The grades 2 and 4 cp-Ti implants used had an identical geometry and microstructure.

## 2. Materials and Methods

### 2.1. Implant Preparation

**Figure 1:**
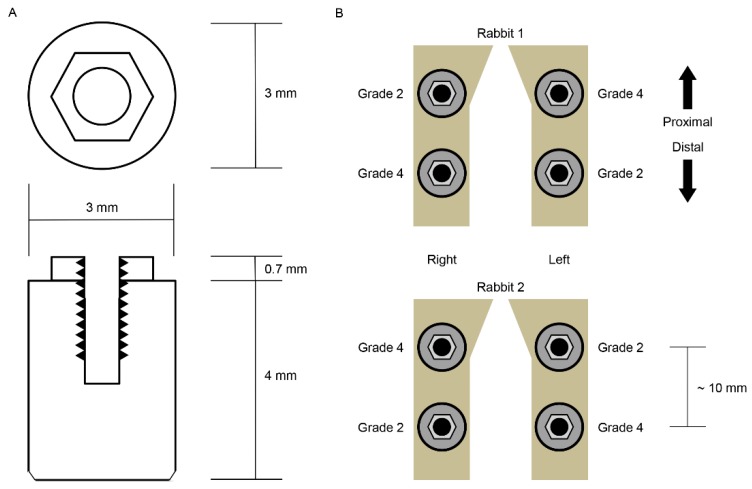

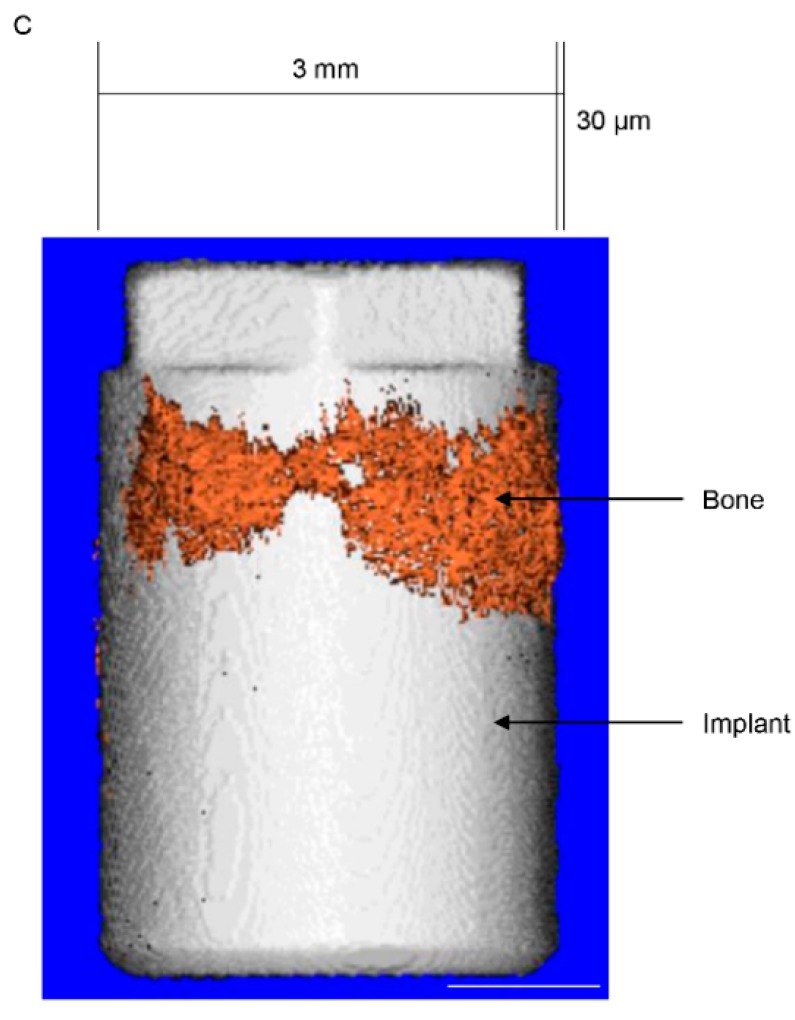
Schematic diagram of the specimens used in this study and the arrangement of the specimens. (**A**) The implant specimens had a simple geometric form: a cylinder that facilitates biomechanical calculations. (**B**) The grades 2 and 4 commercially pure titanium implants were installed into rabbit tibiae according to the Latin Square randomization technique. The distance between the centers of the proximal and distal implants was approximately 10 mm. (**C**) The three-dimensional (3D) bone–implant interfaces were reconstructed via digital image processing. A bone–implant contact surface is shown here. Note that the image of the bone is cut and processed in 30-μm thicknesses.

Twenty-six experimental implants (Deep Implant System, Seongnam, Korea) were prepared, composed of a different grade of cp-Ti: Grade 2 and 4 (*n* = 13 for each grade). All the implants were conventional external hex designed, 3.0 mm in diameter, and 4.0 mm in length. The implants were formed without thread and surface modification: Solid rod or cylindrical design and turned surface (Figure 1A).

### 2.2. Surface Characteristics Analysis

Nine cp-Ti grades 2 and 4 implants were used in the surface character analysis test. Three surface analysis tests were performed for implants of each cp-Ti grade: Field emission scanning electron microscopy (FE-SEM), X-ray photoelectron spectroscopy (XPS), and confocal laser microscopy (CLSM). The FE-SEM (model S-4700, Hitachi, Tokyo, Japan) was used to capture several scaled images of each implant surface (*n* = 3). XPS (Sigma Probe, Thermo Fisher Scientific, Waltham, MA, USA) was used to identify the elemental content and quantify the atomic concentration of the tested surfaces; measurements were repeated three times for each specimen (*n* = 3). CLSM (LSM 800, Carl Zeiss AG, Oberkochen, Germany) was used to measure the surface topographical features of implant sides on three different areas (measurement area: 150 μm × 150 μm on a 200 × optically- and 3 × digitally-magnified image) for each specimen (*n* = 3). The images were filtered using a Gaussian low-pass filter with a cut-off wavelength of 80 µm. The average surface deviation (Sa) and developed surface area ratio (Sdr) were measured.

### 2.3. In Vivo Implant Surgery and Euthanasia

The animal study was approved by the Ethics Committee of the Animal Experimentation of the Institutional Animal Care and Use Committee (CRONEX-IACUC 201702001; Cronex, Hwasung, Korea). All the animal study procedures, including animal selection, management, preparation, and surgical protocols, were performed according to the guidelines of Animal Research: Reporting In Vivo Experiments (ARRIVE) [7]. We installed grades 2 and 4 cp-Ti implants in two male New Zealand white rabbits, each weighing 2.5 to 3.0 kg and aged about six months. They showed no sign of disease or illness before the experiment. Two implants were installed in each rabbit tibia using a standard Latin Square design (Figure 1B). Prior to surgery, the rabbits were anesthetized with an intramuscular injection of tiletamine/zolazepam (15 mg/kg; Zoletil 50, Virbac Korea, Seoul, Korea) and xylazine (33 mg/kg; Rompun, Bayer Korea Ltd., Seoul, Korea). The animals received an intramuscular injection of 33 mg/kg Cefazolin (Yuhan, Seoul, Korea), a preoperative prophylactic antibiotic. The skin of each rabbit’s proximal tibia area was shaved with an electric shaving machine and sterilized with povidone iodine solution, and local anesthetic, lidocaine (1:100,000 epinephrine; Yuhan, Seoul, Korea), was injected into each surgical site. The skin was incised with a surgical blade, and full-thickness periosteal flap reflection was performed to expose each tibia. The implant preparation drilling was conducted on the flat surface of the tibia using a 3-mm-diameter final dental implant drill under simultaneous sterile saline irrigation. The implant was inserted into the drill hole so that the top of the implant was 0.5 mm above the upper cortex of the rabbit tibia without contacting the lower cortex using a silicone ring. Four implants were installed into each rabbit—two grade 2 cp-Ti implants and two grade 4 cp-Ti implants—in a Latin Square design (2 × 2 Latin Square, *n* = 4). After the insertion of the implants, the surgical sites were sutured layer by layer. We allowed a relatively long healing period to present similar bone–implant contacts for the two different types of implants. Rabbits were sacrificed after six weeks of bone healing by intravenous administration of potassium chloride following anesthetization. The implants were exposed by full thickness periosteal elevation and retrieved en bloc with the adjacent bone collar.

### 2.4. Measurement of Three-Dimensional Bone–Implant Contact Area

CT imaging of the harvested implants and bone was performed using a Quantum GX μCT imaging system (PerkinElmer, Hopkinton, MA, USA), located at the Korea Basic Science Institute (Gwangju, Korea). The X-ray source was set to 90 kV and 88 μA with a 10 mm field of view (voxel size = 20 μm; scanning time = 57 min). The CT data were visualized using the Quantum GX’s three-dimensional (3D) viewing software. 3D images of the implant specimens and bone growth were constructed. Image processing for the calculation of the bone–implant contact area was described in a previous study [8]. Briefly, following scanning, the images were segmented using Analyze software version 12.0 (AnalyzeDirect, Overland Park, KS, USA) and filtered to reduce imaging noise. Then, the dataset was manually reoriented using Analyze software to visualize standard coronal, sagittal, and horizontal planes through the implants. The images were reformatted to cubic volume (3D) with a resliced 30-μm image thickness of the surface area on the implant outer surface for the cross-sectional and longitudinal axes. The segmentations of implant and bone-growth images were also performed on Analyze software. As such, 3D rendering of the implants and bone growth was completed. To determine the bone volume on the implant surface in 30-μm thicknesses, the original scanned images were rotated by 10 degrees and the segmentations and 3D rendering were repeated. These 18 repetitions produced the overall 3D bone growth image on the implant surface in 30-μm thicknesses (Figure 1C). The 3D BIC area was the bone formation area on the cylindrical surface of the implant. Assuming that the volume in 30-μm thicknesses was homogeneously filled with bone, the 3D BIC area was determined by dividing the bone volume by the thickness (30 μm).

### 2.5. Measurement of Shear Bond Strength of Bone and Implant

After CT scanning, implant removal torque values were measured within 24 h. The experimental implants had no thread and no fixation via screw action. The constant-speed counter-clockwise rotation of the implant or collar bone results in the disintegration of the BIC or shear bond failure. This continuous removal torque test produces accurate and uniform results [9,10]. Implant removal torque was measured with a motorized torque test stand (TSTM, Mark-10 Co., Long Island, NY, USA). The rotation speed was 0.3 rpm for the lowest angular speed to ensure peak implant removal torque was not skipped between sampling intervals (65 samples/s). The peak implant removal torque was selected from the time–torque curve data. The shear bond strength (MPa) between the bone and osseointegrated dental implant, or binding force per unit area in this study, was calculated by dividing the peak implant removal torque (Ncm) by the determined 3D BIC area (mm^2^) and the implant radius (1.5 mm). The units were adjusted before calculation.

### 2.6. Statistics

The independent t-test was used to determine the statistical significance of the surface roughness parameters, Sa and Sdr, between the grade 2 and 4 cp-Ti implants. The paired t-test was used to compare the 3D BIC area and the shear bond strength between the groups. All data were evaluated at the significance level of 0.05.

## 3. Results

### 3.1. Surface Characteristics Analysis

**Figure 2:**
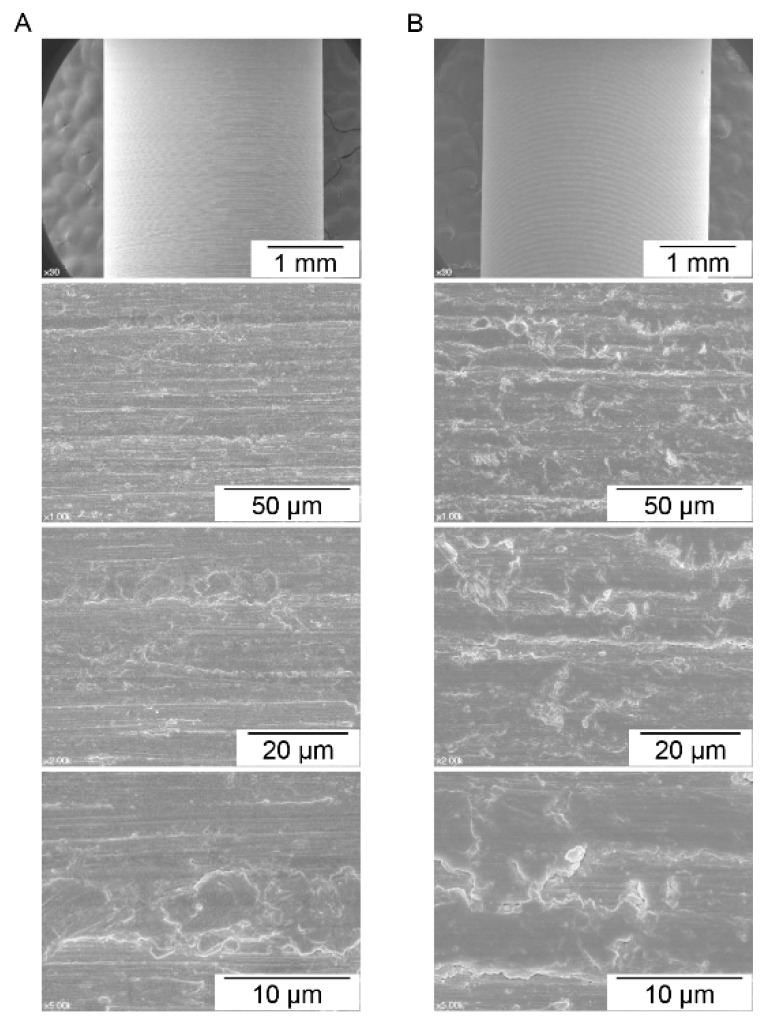
Field emission scanning electron microscopy (FE-SEM) images of commercially pure titanium of (**A**) grade 2 and (**B**) grade 4. Many machining grooves were observed. Similar surface characteristics were found for both grades, which implies that the influence of topographical features on the biological response should not be different between the commercially pure titanium grades used in this study.

The FE-SEM image of each surface area is shown in Figure 2. The grades 2 and 4 cp-Ti surfaces show similar surface characteristics, which resulted from the computer numerical control machining manufacturing process. The results of the CLSM analysis showed that the means of Sa were 0.49 ± 0.082 μm for grade 2 cp-Ti implants and 0.45 ± 0.088 μm for grade 4 cp-Ti implants. No significant difference was found for Sa between the Ti grades (*p* = 0.63). The means of Sdr were 16.9% ± 1.5% for the grade 2 implant group and 14.4% ± 2.1% for the grade 4 implant group, and no significant difference was found between the groups (*p* = 0.18). These results indicate that the grades 2 and 4 cp-Ti implants used in this study had similar surface topographical features. However, surface chemistry depended on the Ti grades. The XPS showed that the atomic composition of the grade 2 cp-Ti surface was significantly different from that of the grade 4 surface (Table 1).

### 3.2. Three-Dimensional Bone–Implant Contact Area and Shear Bond Strength

The bone–implant contact surface area and three-dimensional bone–implant contact ratio are summarized in Table 2. The means and standard deviations of the contacted surface area for grades 2 and 4 cp-Ti implants were 12.7 ± 1.2 mm^2^ and 11.5 ± 1.6 mm^2^, respectively. The 3D bone–implant contact percentages of grades 2 and 4 cp-Ti implants were 33.6% ± 3.2% and 30.5% ± 4.3%, respectively. Neither the contacted surface area nor the 3D bone–implant contact showed any statistical difference between the two grades. The peak implant removal torques of grades 2 and 4 cp-Ti implants were 2.9 ± 0.4 Ncm and 1.9 ± 0.3 Ncm, respectively. The shear bond strength of grade 2 cp-Ti implants was 1.5 ± 0.2 MPa, which was statistically significantly higher than that of the grade 4 cp-Ti implants, which was 1.1 ± 0.1 MPa (*p* = 0.032) (Table 2).

## 4. Discussion

The grades 2 and 4 cp-Ti implants used in this study showed similar surface topographic features in SEM images and CLSM analysis. The grade 2 cp-Ti implants showed higher shear bond strength than the grade 4 implants despite the similar 3D bone–implant contact ratios between these two grades. Considering the significant differences in the compositions between the grades 2 and 4 cp-Ti surfaces, the bone response was evaluated to be stronger to grade 2 than to grade 4. The results of this study suggest that the bone would have its own affinity, depending on the materials, and that an actual bond would exist in addition to the physical contact and friction between bone and an implant surface [3]. Although further verification studies are required, the osseointegration phenomenon of a Ti dental implant appears to be a bioaffinitive response to the Ti surface beyond the bony isolation.

The pull-out test of integrated implants has been widely adopted to test osseointegration strength [11,12,13,14,15]. However, we used rotating force instead of pull-out force because the removal direction for the pull-out test could be nonparallel to the long axis of the implant, imposing unintentional lateral force on the implant, which can introduce measurement error. This adverse effect was minimized in this study by using rotational force for implant removal and a motorized torque test stand to simplify adjustment to the long axis [10].

This study compared the grades 2 and 4 implants using the rabbit tibia model. Sample size determination and randomization are important to reduce the number of sacrificed animals [7]. Following the guidelines of the 3Rs (replacement, reduction, and refinement) of ARRIVE, this study used the 2 × 2 Latin Square design, which minimized the sample size and accomplished complete randomization in the arrangement of the implant groups [7]. Considering the standard deviation of the 3D BIC area and ratio measures, the number of sacrificed animals (two) was estimated to be adequate although sample size calculation was not performed in this study, which would require more animals than two.

Many studies reported significant differences in the amount of bone–implant contact when implant surfaces were topographically changed [16]. Further studies are needed to evaluate the effects of surface topography on the quality of osseointegration. A roughened surface increases the bone–implant area, increasing the physical interlocking between bone and the surface, which would result in the same shear bond strength or removal torque per unit area when the implant surfaces, topographically changed or not, have the same chemical composition. Conversely, the topographical change could have a qualitative influence on the bone–implant contact, which remains to be verified. In addition, further studies are required to evaluate osseointegration qualities by comparing the bone–implant contact and shear bond strength at the different phases of bone healing after implant placement.

## 5. Conclusions

Grades 2 and 4 cp-Ti surfaces with similar topographical features showed different bond strengths in the bone–implant contact. Considering the results of this study, an actual bond may occur between bone and a Ti dental implant surface beyond the physical attachment of bone to the surface.

## Figures and Tables

**Table 1:** The atomic composition of surfaces of grades 2 and 4 commercially pure titanium (cp-Ti) obtained by X-ray photoelectron spectroscopy (XPS).

Element	Cp-Ti Grade 2	Cp-Ti Grade 4	*p*-Value
Carbon (C)	3.66% ± 0.21%	4.03% ± 0.39%	2.3 × 10^−1^
Oxygen (O)	1.15% ± 0.10%	3.21% ± 0.29%	3.0 × 10^−4^ *
Iron (Fe)	0.26% ± 0.02%	0.39% ± 0.05%	2.0 × 10^−2^ *
Titanium (Ti)	94.93% ± 0.32%	92.38% ± 0.13%	2.1 × 10^−4^ *

* Statistically significant.

**Table 2:** Three-dimensional bone-to-implant contact (BIC) area and shear bond strength.

	Grade 2 cp-Ti	Grade 4 cp-Ti	*p*-Value
3D BIC area (mm^2^)	12.7 ± 1.2	11.5 ± 1.6	0.35
3D BIC ratio (%)	33.6 ± 3.2	30.5 ± 4.3	0.35
Implant removal torque (Ncm)	2.9 ± 0.4	1.9 ± 0.3	0.052
Torque per unit (Ncm/cm^2^)	23.2 ± 3.5	16.5 ± 1.0	0.032 *
Shear bond strength (MPa)	1.5 ± 0.2	1.1 ± 0.1	0.032 *

* Statistically significant.
